# Approaches and Challenges for Biosensors for Acute and Chronic Heart Failure

**DOI:** 10.3390/bios13020282

**Published:** 2023-02-16

**Authors:** Sariye Irem Kaya, Ahmet Cetinkaya, Goksu Ozcelikay, Seyda Nur Samanci, Sibel A. Ozkan

**Affiliations:** 1 Department of Analytical Chemistry, Gulhane Faculty of Pharmacy, University of Health Sciences, Ankara 06018, Turkey; 2 Department of Analytical Chemistry, Faculty of Pharmacy, Ankara University, Ankara 06560, Turkey; 3 Department of Analytical Chemistry, Graduate School of Health Sciences, Ankara University, Ankara 06110, Turkey

**Keywords:** heart failure, biomarkers, detection, biosensors, diagnosis

## Abstract

Heart failure (HF) is a cardiovascular disease defined by several symptoms that occur when the heart cannot supply the blood needed by the tissues. HF, which affects approximately 64 million people worldwide and whose incidence and prevalence are increasing, has an important place in terms of public health and healthcare costs. Therefore, developing and enhancing diagnostic and prognostic sensors is an urgent need. Using various biomarkers for this purpose is a significant breakthrough. It is possible to classify the biomarkers used in HF: associated with myocardial and vascular stretch (B-type natriuretic peptide (BNP), N-terminal proBNP and troponin), related to neurohormonal pathways (aldosterone and plasma renin activity), and associated with myocardial fibrosis and hypertrophy (soluble suppression of tumorigenicity 2 and galactin 3). There is an increasing demand for the design of fast, portable, and low-cost biosensing devices for the biomarkers related to HF. Biosensors play a significant role in early diagnosis as an alternative to time-consuming and expensive laboratory analysis. In this review, the most influential and novel biosensor applications for acute and chronic HF will be discussed in detail. These studies will be evaluated in terms of advantages, disadvantages, sensitivity, applicability, user-friendliness, etc.

## 1. Introduction

Cardiovascular diseases (CVDs) is a broad term covering heart and circulatory diseases and is listed in the literature as the leading cause of death worldwide. Management of CVDs, such as heart failure, hypertension, stroke, coronary heart disease, etc., is a huge economic burden for societies due to high incidence and mortality rates. Although the risk factors and underlying mechanisms for CVDs differ, advancing age, life and dietary habits, and genetic factors are the mainly evaluated parameters [1,2,3]. Among these diseases, heart failure (HF) has a significant place as a disease with increasing prevalence and is not easy to identify [4,5]. In its broadest definition, HF is the inability of the heart to adequately pump the blood necessary for the body [6]. Fatigue and exertional dyspnea are the most common symptoms of HF, and it is a complex clinical phenomenon that may be caused by functional or structural problems of the heart [7,8]. Although its symptoms and definition may seem simple, identifying and managing the presence and causes of HF can be challenging [7]. It is also possible to classify HF as acute and chronic. Acute HF is characterized by the onset of severe symptoms that require immediate treatment, while stable and persistent symptoms characterize chronic HF. Appropriate treatment approaches are offered for both [7].

**Figure 1:**
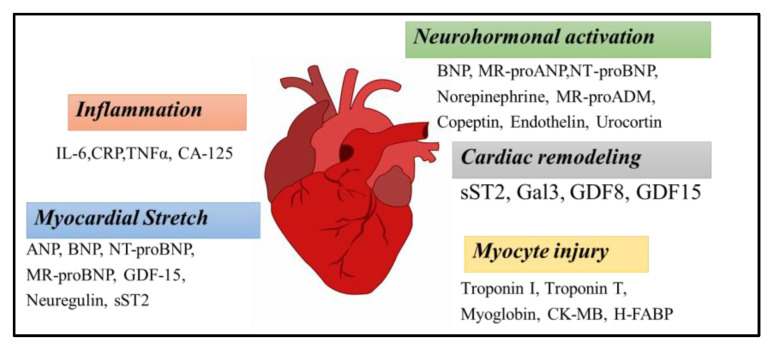
Overview of the heart failure (HF) biomarkers.

Identification, prediction, and diagnosis of HF is a compelling process; therefore, it is important to evaluate the clinical characteristics in terms of diagnosis and prognosis [4,8]. Biomarkers are analytes that can be detected in the blood as indicators that give an idea about the processes in the body [5,9]. Therefore, when it was evaluated from the point of biomarkers, HF management by enlightening biological pathways related to HF pathogenesis can be very useful [4,8]. Acute/chronic heart failure biomarkers (Figure 1) can be defined according to the mechanisms with which they are associated. The main groups are as follows: inflammation (tumor necrosis factor α [TNFα], growth differentiation factor 15 [GDF 15], ST2, etc.), neurohormonal therapy (renin, copeptin, endothelin-1, etc.), extracellular matrix remodelling (galectin-3 [GAL-3], interleukin-6 [IL-6], etc.), oxidative stress (myeloperoxidase [MPO], oxidized LDL, urinary biopyrrins, etc.), myocyte stretch (GDF-15, soluble ST2 [sST2], B-type natriuretic peptide [BNP], etc.), myocardial injury (troponin T and I, heart type FA binding protein [hFABP], and myosin light-chain kinase, etc.) [10]. Additionally, it is possible to classify biomarkers in terms of HF disease management. For example, some of them can be useful for diagnosis (BNP, miRNA, sST2), monitoring prognosis (troponin, BNP, GAL-3, IL-6, TNFα), or evaluating the severity of the disease (copeptin, sST2, CRP, hFABP) [4,8,11,12,13].

Selective, rapid, and sensitive determination of biomarkers that can be beneficial at every stage of HF management is also very critical. For this purpose, that the aim is for the biosensors to help with early-stage diagnosis and effective monitoring of the disease prognosis for HF by targeting the most suitable biomarkers [2,14]. The most significant advantages of biosensors are that they are rapid, reliable, and portable, and offer real-time analysis, high selectivity, and sensitivity compared to other available methods in the literature [15]. Studies in the field of biosensors are both very popular and extensive. In addition, they continue to evolve. When the literature is examined, there are publications that compile biosensor studies for the determination of biomarkers associated with cardiovascular diseases such as HF [5]. For example, Ouyang et al. [2] reviewed point-of-care biosensors for cardiovascular blood biomarkers. Pourali et al. [16] evaluated the voltammetric biosensors for cardiac troponins. On the other hand, Azzouz et al. [17] examined nanomaterial-based aptasensors for cardiovascular diagnostic applications in their study.

To the best of our knowledge, no studies in the literature solely focus on biosensor applications for acute/chronic HF biomarkers. This current review aims to be useful to researchers by filling this gap and evaluating the HF biomarkers for diagnosis and therapy and the biosensor applications. It will also offer perspective on future approaches, such as wearable biosensors.

## 2. Heart Failure Biomarkers for Diagnosis and Therapy

Heart failure is a global pandemic that affects ~26 million people worldwide. Age, genetic status, gender, environmental risk effects, smoking habits, hypertension, coronary artery disease, atrial fibrillation, and diabetes trigger the formation of the disease [18].

HF occurs when the heart cannot pump enough blood to meet metabolic needs. If the left ventricle does not work enough, systolic HF and diastolic HF appear. The left ventricle cannot squeeze strongly sufficient in systolic HF, and therefore becomes stiff in diastolic HF. Right-sided HF can occur when the left ventricle is damaged due to a change in blood flow. Too much blood can collect in one place and cause swelling in the legs or abdomen. HF was evaluated as acute and chronic HF. Acute HF occurs very suddenly and requires immediate intervention. Chronic HF is observed gradually over time. HF symptoms are described as breathlessness, fast/irregular heart rhythm speed, weakness, nausea, and pain. The left ventricular ejection fraction (LVEF) leads the way in HF. According to the American Heart Association, LVEF levels should be within the range of 50% to 75% [19].

The development and progression of HF result from many complex interactions of cardiovascular diseases that lead to neurohormonal activation, cardiac remodeling, inflammation, myocardial stretch, and myocyte injury [20].

A biomarker is an indicator of the normal biological process, pathogenic processes, or pharmacological responses to a therapeutic intervention. An ideal biomarker should be non-invasive, low-cost, and easily reproducible, and should offer sensitivity and specificity. Many biomarkers have been identified for the clinical diagnosis of heart failure [20]. They have an important role in the diagnosis, evaluation of risks, assessment of prognosis, and monitoring of the response of the therapy. Protein-based HF biomarkers were classified into several diseases. The inflammatory disease biomarkers are mostly known as interleukin (IL-6), C-reactive protein (CRP), tumor necrosis factor-alpha (TNFα), and cancer antigen 125 (CA-125) [21]. Troponin-I (cTnI) and -T(cTnT), myoglobin, Creatine Kinase-MB (CK-MB), and heart-type free fatty acid binding protein (H-FABP) take place in a group of myocyte injury biomarkers [22]. Soluble suppression of tumorigenesis-2 (sST2), galectin-3 (Gal3), growth differentiation factor 8 (GDF8), and growth differentiation factor 15 (GDF15) biomarkers belongs to cardiac remodeling. The B-type natriuretic peptide (BNP), mid-regional pro-atrial natriuretic peptide (MR-proANP), N-terminal pro-B-type natriuretic peptide (NT-proBNP), norepinephrine, mid-regional pro-adrenomedullin (MR-proADM), copeptin, endothelin, and urocortin all fall under the neurohumoral markers group. Atrial natriuretic peptide (ANP), B-type natriuretic peptide (BNP), NT-proBNP, growth differentiation factor-15 (GDF-15), Neuregulin, and sST2 were found in the myocardial stretch [23].

BNP and NT-proBNP are referred to as gold-standard biomarkers in many published papers because they are used in the diagnosis of HF and contribute to the standard clinical HF diagnosis [24]. The NT-proBNP level in patients with acutely decompensated HF (108 pg/mL) is 23 times higher than without HF (4.639 pg/mL). The cut-off values of NT-proBNP and BNP were reported as 900 pg/mL and 100 pg/mL, respectively [18]. Norepinephrine levels increase above 393 pg/mL in the presence of HF [20]. Troponin, especially cTnI and cTnT, can be a sensitive and specific indicator of acute myocardial infarction [25]. Troponin protein releases into the bloodstream within 1–3 h when there is damage to the heart muscle. The troponin level reaches 100 ng/mL and remains the same for 10 days. cTn can be measured in saliva and urine instead of blood serum. sST2 is a novel biomarker related to HF. However, this biomarker is not only used for the diagnosis of HF but also in the diagnosis of lung disease [25]. The clinicians can better interpret the mortality associated with HF when combining sST2 data with NTproBNP data. Gal-3 is a stable biomarker with no relation to age, BMI, or gender. The cut-off of the Gal-3 value is established as 10.1 ng/mL and 160 pg/mL with 77.78% of sensitivity and 95% specificity [26]. The increase in IL6 level indicates the risk of heart failure with preserved ejection fraction (HFpEF) over time [21]. Typical cardiac biomarkers consisting of CRP, TNFα, CA-125, cTnI, cTnT, myoglobin, and CK-MB have an important role in diagnosing myocardial infarction (AMI) [27]. When a heart attack occurs, the CK-MB level remains high for the first 18–24 h and gradually returns to its normal level after a few days [28]. The elevated CRP levels may reflect the inflammatory activity of AMI. A C-reactive protein value of >10 mg/L is considered a risk for cardiovascular prevention. The cut-off of plasma H-FABP level is 4 ng/mL when acute coronary syndrome is suspected [20].

Biomarkers and other investigations contribute to the diagnosis of HF. Chest X-ray and echocardiography are generally used as traditional clinical diagnosis techniques. However, echocardiogram testing can be difficult to perform in an emergency [29]. In addition, the chest X-ray can create an issue regarding sensitivity and specificity. HF biomarkers can be detected using the Enzyme-linked Immunosorbent Assay (ELISA) method. However, a point-of-care (POC) rapid quantitative analysis has come to the fore in recent years because clinicians profit from this useful method to monitor the prognosis of patients with HF [30].

Molecular recognition mechanisms are based on the lock and key model, with the substrate and the receptor showing specific interactions without being ignored by complementary geometric shapes that fit into one another [31]. The enzyme-substrate, antibody-antigen (Ab-Ag), and receptor-effector mechanisms can be integrated into many electrochemical biosensor studies. For example, aptasensors, immunosensors, enzyme-based biosensors, and molecularly imprinted polymer-based biosensors are widely used to determine HF biomarkers in biological samples [32].

## 3. Applications of Biosensors for Acute and Chronic Heart Failure

The numerous electrochemical studies and biological detection of biomarkers often utilized in acute and chronic HF have been extensively examined over the past five years. When the studies in the literature were examined, primarily biosensor-based approaches were discussed. In addition, while reviewing recently published articles on diverse aspects of electrochemical techniques, there is substantial research into the application of electrochemical methods to detect acute and chronic heart failure biomarkers. In addition, Table 1 presents detailed information on various electrode modification approaches as well as electrochemical biosensing of acute and chronic HF biomarkers.

**Figure 2:**
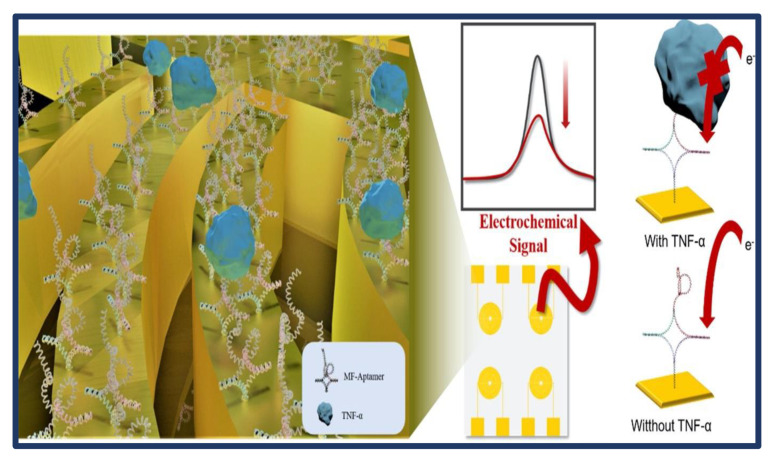
Schematic representation of fabricated tumor necrosis factor-α (TNF-α) sensing biosensor. CV was carried out for 10 cycles within the potential range of −0.4 V to 0.4 V with a scan rate of 0.1 V/s. Reprinted from Ref. [38] with permission from Elsevier.

Among the papers in Table 1, some of the studies that stand out with different methods and materials are discussed here. Kim et al. [38] developed a brand new ITO platform for detecting inflammatory biomarkers using multifunctional DNA constructs. The MF-Aptamer exhibited properties such as recognizing TNF-α, being immobilized on a substrate, and providing an electrochemical peak (Figure 2). Furthermore, the MF-Aptamer could be assembled simply by annealing at 80 °C for 5 min. With TBM PAGE, it was shown that the desired structure was formed without losing the functional ability of the DNA fragment. Ag+ was used as a redox species in the proposed MF-Aptamer in this study. The limit of detection (LOD) of the suggested biosensor was established as 0.07 pg/mL, and TNF-α could be obtained in human serum samples across a dynamic range of 0.15 pg/mL to 15 ng/mL. Commercially available TNF-α detection kits usually have a detection limit of 1.7 pg/mL. However, since the LOD obtained with this developed sensor is 0.14 pg/mL, it is very sensitive compared to TNF-α detection kits available in the market. In addition, having a wide linear working range is an important advantage of the modified sensor. Consequently, the electrochemical determination technique utilizing the MF-Aptamer described in this paper offers a new platform for identifying particular biomarkers in human serum.

**Figure 3:**
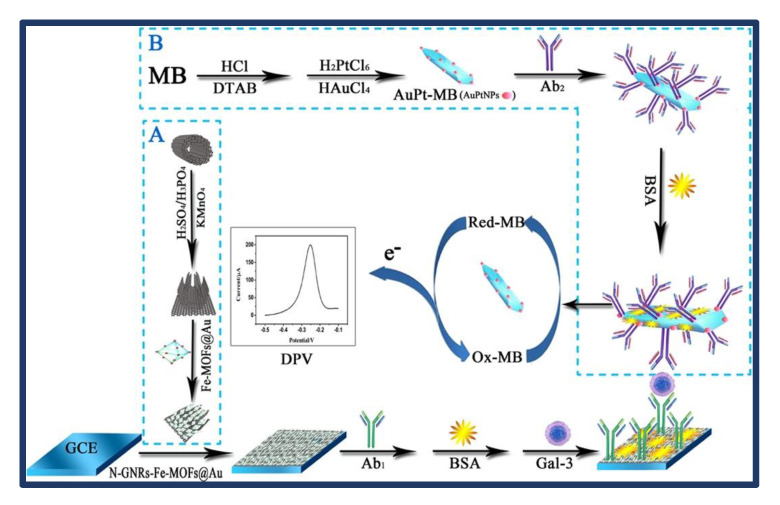
Schematic representation of the electrochemical immunosensor interfaces’ step-by-step assembly process. The methods used to prepare the bioconjugates AuPt-MB-Ab2 and N-GNRs-Fe-MOFs@AuNPs in (**A**) and (**B**), respectively. The differential pulse voltammetry (DPV) scan was performed from −0.5 V to −0.1 V with a pulse amplitude of 50 mV, pulse width of 50 ms, and pulse period of 100 ms. Reprinted from Ref. [44] with permission from Elsevier.

In this study by Tang et al. [44], an effective sandwich-type electrochemical immune sensor based on N-GNRs-Fe-MOFs@AuNPs and AuPt-MB nanocomposites was fabricated to detect Gal-3. The substrate platform of the immunosensor, N-GNRs-Fe-MOFs@AuNPs, has a fairly large specific surface area and many active sites that can capture primary antibodies and accelerate electron transfer. AuPt-MB is a new redox nanoprobe that can easily collect detecting antibodies via Pt-NH2 and Au-NH2 and generate and amplify the electrochemical signal (Figure 3). The intended immunosensor has superior sensitivity and a larger linear range for the detection of Gal-3 when compared to the analysis performance of existing methods. The developed immunosensor showed excellent sensitivity, reproducibility, stability, and selectivity against Gal-3, and the obtained values were promising for possible use in clinical diagnosis. However, this sensor was disadvantageous for point-of-care detection in clinical applications as it was time-consuming to create the entire sensor.

**Figure 4:**
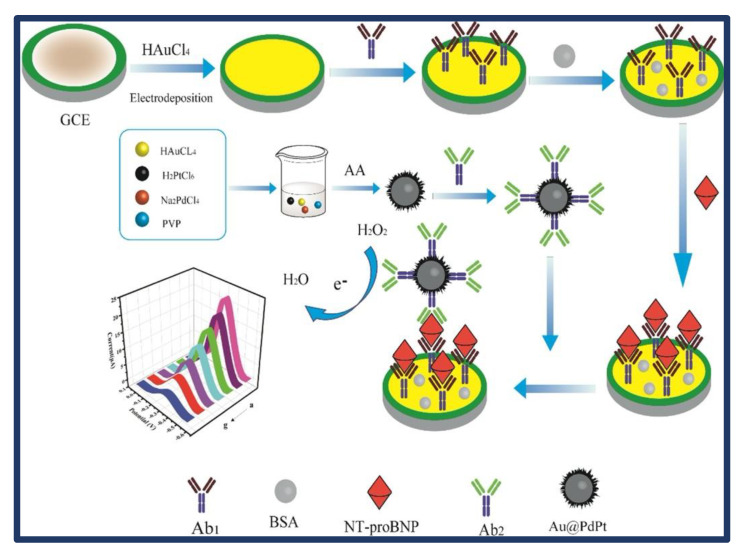
Diagrammatic representation of the sandwich electrochemical immunosensor preparation process. By square-wave voltammetry, the prepared electrode was scanned 0.0 V−0.6 V in 10 mL PBS (pH 7.4) involving 5 mM H_2_O_2_. Reprinted from Ref. [45] with permission from Elsevier.

Zhang et al. [45] fabricated an NT-proBNP sandwich electrochemical immunosensor using electroplated Au NPs as substrate material and Au@PdPt RTNs as current signal amplifiers. Due to their high specific surface area and good conductivity, Au NPs were able to immobilize Ab1 successfully. Au@PdPt RTNs served as effective signal probes and signal amplifiers, thanks to their large specific surface area to load Ab2 and their outstanding catalytic capabilities for H_2_O_2_ reduction (Figure 4). The immunosensor showed a wide detection range of 0.1 pg/mL to 100 ng/mL and a LOD of 0.046 pg/mL, thanks to the helpful collaboration of Au NPs and Au@PdPt RTNs. An interfering experiment and the examination of spiked samples further confirmed the feasibility of this procedure. As a result, the proposed immunosensor might have future use in the clinical diagnosis of NT-proBNP.

**Figure 5:**
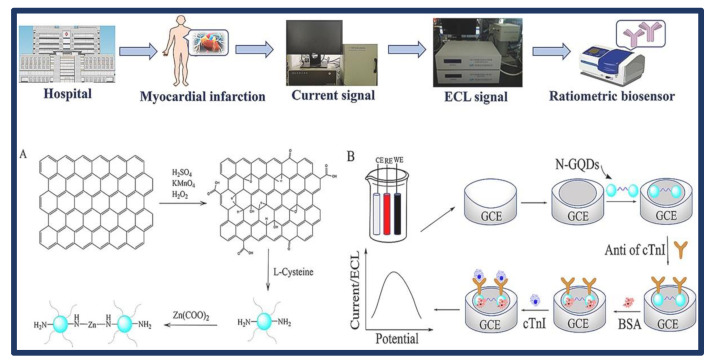
Schematic of the prepared rout of N, Zn-GQDs (**A**) and the ratiometric biosensor for analysis of cardiac troponin I (**B**). The electrochemical parameter was −0.6–0.8 mV, the scanning rate was 0.1 V/s, the condition of electrochemiluminescence (ECL) intensity was the scanning range of −2.0−0 V in 0.05 M K_2_O_8_S_4_ solution and the potential increment of 10 mV. Reprinted from Ref. [67] with permission from Elsevier.

A ratiometric immunosensor with excellent sensitivity and reliability for the detection of cTnI using N, Zn-GQDs as signal amplification with ECL and DPV signals was developed by Liu et al. [67]. For signal amplification, GO provided a huge surface area that could be loaded with a large number of N, Zn-GQDs. The amide bond between the N, Zn-GQDs, and the cTnI antibody allowed for effective modification of N and Zn-GQDs on the surface of the GCE. The specificity between antigens and antibodies allowed for the identification of cTnI. (Figure 5). The results showed that N, Zn-GQDs are highly sensitive to and precise for the ratiometric immunosensor. In addition, the developed sensor’s linear operating range and LOD values were found to be 10–106 pg/L and 4.59 pg/L, respectively. The new method was tested for the detection of cTnI in human serum, and the findings showed good agreement with the reference values obtained by ELISA with a 9.09−11.1% RSD. It was demonstrated that it might be used for the more sensitive and extensive detection of cTnI in human serum samples. In addition, the ratiometric immunosensor based on N,Zn-GQDs can also be used as an alternative application for identifying other biomarkers.

**Figure 6:**
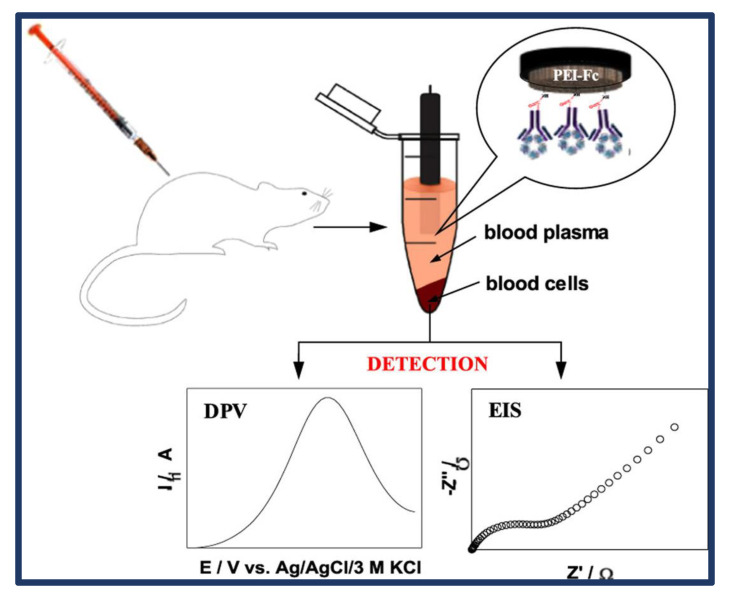
Schematic representation of the production of an electrochemical C-reactive protein (CRP) immunosensor. Reprinted from Ref. [53] with permission from Elsevier.

A highly selective biosensing platform functionalized with ferrocene residues for the electrochemical detection of C-reactive protein in blood samples was created by Kowalczy et al. [53]. Although it is well known that the C-reactive protein is not electrochemically active, it added ferrocene moieties to the PEI network via an unstable carbon linker, enabling this polymeric material to be used as a redox detector. In addition, the PEI network on the electrode surface performed the covalent binding of an antibody in the optimal direction for antigen-antibody recognition (Figure 6). The dispersed quantum crystal microbalance measurements were used to confirm the orientation of the antibody molecules in the recognition layer. The electrochemical CRP immune(bio)sensor shown was able to discriminate with its high selectivity, sensitivity, and wide linearity range of 1 to 5 × 104 ng/mL. The developed technology was effectively applied to measure the level of CRP in real blood samples (rat model). The PEI-Fc-Ab layer-based CRP immuno(bio) sensor is expected to offer significant potential in medical diagnostics.

**Figure 7:**
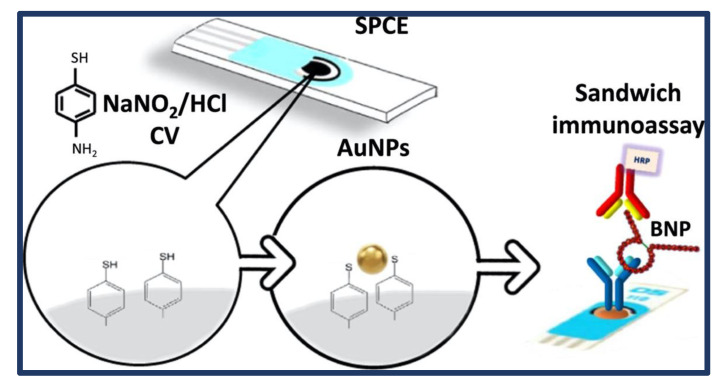
Schematic representation of fabricated B-type natriuretic peptide (BNP)-sensing biosensor. Reprinted from Ref. [64] with permission from Elsevier.

By combining a sandwich immunoassay with an HRP-labeled detector antibody and SPCEs transduction platforms built upon electrode modification with AuNPs, a disposable electrochemical-based immunosensor for the measurement of the BNP was created by Serafin et al. [64]. Figure 7 summarizes the steps involved in the preparation of the electrodes modified by grafting of 4-aminothiophenol and further attachment of AuNPs. Then, 1 mM NaNO_2_ was slowly added to the 1 mM 4-aminothiophenol solution prepared in a cold environment using 0.5 M HCl with continuous stirring. SPCE was then immersed in an electrochemical cell BNP determination and performed using CV between 0.6 and 1.0 V at 100 mV s^−1^. The analytical performance of this AuNPs-nanostructured immunosensing scaffold is superior to that of immunosensors made using other AuNPs modification techniques. The LOD value obtained by the immunosensor is 100 times lower than the clinical cut-off value determined in serum for heart failure patients. Additionally, it has been shown that the immunosensor is suitable for precisely measuring the biomarker in human sera with a little sample treatment (only a 10 times dilution), with results that are in good agreement with those achieved by utilizing a traditional ELISA methodology. This new amperometric immunosensor’s promising analytical performance, ease of use, disposability, and the ability to use portable electrochemical transducers make it a very appealing alternative to widely used ELISAs for the development of automated POC systems for on-site detection of this HF disease biomarker.

## 4. Future Prospects

The survival rate is still low in HF patients, demonstrating the importance of early diagnosis with the determination of biomarkers. Regular measurement and monitoring of significant biomarkers can assist in the care of HF for patients [12,86]. The future of biosensor technologies in healthcare is now going towards small, digital, and mobile devices. In this way, the aim is to make it possible to collect data from patients without them having to apply to the hospital or clinic. Various parameters related to HF can be monitored with wearable devices placed on clothes or used as accessories. Occurrence of atrial fibrillation, detection of daily activity levels, or daily steps can provide beneficial outcomes for HF management and monitoring [87]. Additionally, especially in asymptomatic patients, by evaluating rapidly changing physiological information with the assistance of digital biomarkers and artificial intelligence, the prediction of situations that may be encountered in HF offers a new approach [4].

In addition to all these, the aim is to improve certain features of traditional biosensor devices and make them more useful. Researchers continue to work on objectives such as miniaturization, portability, less sample requirement, and simplification of the measurement process.

## 5. Conclusions

HF is a significant type of CVD that requires a troublesome identification, prediction, and diagnosis process. Therefore, the selection, evaluation, and detection of significant biomarkers play a key role in disease management. In this review study, novel approaches to electrochemical biosensors for acute and chronic HF were explained by evaluating the most recent works in the literature. It was found that TNFα, CRP, cTnI, and miRNA are mainly studied analytes in electrochemical biosensors. To improve the properties of electrochemical biosensors, such as selectivity and sensitivity, hybrid systems modified with various materials, such as nanomaterials, have been preferred. The most frequently used electrochemical techniques are DPV and EIS. Immunosensors, on the other hand, have been mostly used due to their many advantages. Furthermore, it is seen that the applied samples are mostly biological samples such as human serum. In conclusion, it can be said that the researchers aim to develop sensors that will provide advantages and superiority in clinical and point-of-care applications.

## Figures and Tables

**Table 1:** Recently developed biosensor-based methods for acute and chronic heart failure (HF).

Analyte	Sensor	Technique	Linear Range	LOD	RSD (%)	Real Sample	Recovery	Ref
TNFα	Anti-TNFα/BSA/PAMAM/ NFs-AuE	CV EIS	10–200 pg/mL	669 fg/mL	NR	Human serum Saliva	NR	[33]
TNFα	AuE	ACV	0.1–500 nM	100 pM	NR	Urine Saliva	NR	[34]
TNFα	TNFα/anti-TNFα-Ab_1_/AuNPs/S-MWCNTs/GCE	CV EIS	0.01–1.0 pg/mL	2.00 fg/mL	0.61	Human plasma	100	[35]
TNFα	AuHCF-AuNPs/SPE	DPV	10 pg/mL–40μg/mL	5.5 pg/mL	0.46	Human serum	NR	[36]
TNFα	ITO Electrode	EIS CV	0.02–4 pg/mL	6 fg/mL	NR	Human serum	97.07–100.19	[37]
TNFα	PDMS/AuE- ITO	CV	0.15 pg/mL–15 ng/mL	0.07 pg/mL	NR	Human serum	NR	[38]
TNFα	GPTES-ITO-PET	CV EIS	0.01–1.5 pg/mL	3.1 fg/mL	0.87	Human Serum	96.51–100.90	[39]
TNFα α	AuE (microelectrodes)	CV EIS	NR	1–15 pg/mL	NR	Human Saliva	NR	[40]
IL6 TGFβ1	AuNP-SPE	DPV	10^2^−10^8^ fM 50−10^5^ fM	47.9 fM 16.6 fM	NR	Human DNA	NR	[41]
Uric acid	GCE	DPV CV	10–1000 μmol/L	107 nmol/L	0.3–8.5	Human serum	95.8–104	[42]
Gal-3	MIPs/SPE	CV EIS	0.5−5000 ng/mL	NR	NR	Human Serum	NR	[43]
Gal-3	N-GNRs-Fe-MOFs@AuNPs/GCE	DPV	100 fg/mL−50 ng/mL	33.33 fg/mL	2.75	Human plasma	98.91–104.84	[44]
NT-proBNP	Au@PdPtRTNs/GCE	Amperometry CV EIS	0.1 pg/mL–100 ng/mL	0.046 pg/mL	3–5.4	Human Serum	98.7–101.3	[45]
NT-proBNP	SPE, Pt counter electrode	EIS	0.02–1 pg/mL	0.02 pg/mL	NR	Saliva	99 ± 8	[46]
ST2	GP Electrode	EIS CV	0.1 fg/mL−100 fg/mL	0.124 fg/mL	1.16–15.59	Human Serum	100–113.46	[47]
Cholesterol	ChOx- CSPPy-g-C_3_N_4_H^+^/GCE	CV	0.02–5.0 Mm	8.0 µM	1.8–2.7	Human Serum	97–101	[48]
CRP	SPCE	CV EIS DPV	800,000−0.008 µM	0.92 nM	NR	Leaf	106.11–107.05	[49]
CRP	GCE	CV DPV	10 pg/mL–10 μg/mL	0.44 pg/mL	5.93–12.02	Human Serum	NR	[50]
CRP	BSA/ZnO/MPC/IL/anti-CRP/CPE	EIS DPV	0.01–1000 ng/mL	0.005 ng/mL	<6	Human Serum	94.5–107.0	[51]
CRP	PMPC-SH/SAM/AuNPs/SPCE	DPV	5–5000 ng/mL	1.6 ng/mL	<1.34	Human Serum	NR	[52]
CRP	PEI-Fc /anti-CRP/GCE	DPV EIS	10–50,000 ng/mL	0.5 ng/mL	8.5	Blood sample	NR	[53]
CRP	MB-NH_2_-SWCNT-AuNPs/SPE	CV DPV EIS	5 pg/mL–1 μg/mL	5 pg/mL	>13.38	Blood sample	80	[54]
CRP	Fc-ECG/MEL/AuNPs/SPE	CV EIS DPV	0.001–1000 μg/mL	0.30 μg/mL	6.59	Human Serum	98.69–102.43	[55]
CRP	anti-CRP rGO/Ni/PtN/SPCE	Amperometry	2–100 μg/mL	0.80 μg/mL	8.0	Human Serum	NR	[56]
CRP	MWCNTs/AuE	EIS CV	0.084–0.84 nM	4\0 pM	3.15	Human Serum	NR	[57]
CRP	ERGO/PTyr/	DPV EIS	1.09–100 μg/L	0.375 μg/L	NR	Human Serum	NR	[58]
CRP	BSA/anti-CRP/MPA/Au	CV SWV	5–220 fg/mL	2.25 fg/mL	3.12	Human Serum	NR	[59]
PCT	PCT-Ab-AgNp-SLG/ITO	CV EIS	2–25 ng /mL	0.55 ng/mL	NR	Human serum	NR	[60]
PCT	MoO_3_-Au-rGO-Ab2/GCE	CV	0.01 pg/mL−10 ng/mL	0.002 pg/mL	2.30	BSA	NR	[61]
PCT	g-C_3_N_4_-NiCo_2_S_4_-CNTs-AgNPs /GCE	DPV	0.05–50 ng/mL	16.70 pg/mL	3–5	Human serum	98.40–102.74	[62]
NT-proBNP	Paper Electrode	LASV SWASV	53–590 pM	300.0 pM	NR	Human Serum	NR	[63]
BNP	AuNPs-S-Phe/SPCE	EIS CV	0.014–15 ng/mL	4 pg/Ml	6.4	Human Serum	NR	[64]
BNP	PPIX/N–ZnO NP/ITO	EIS	1 pg/mL–0.1 μg/ mL	0.14 pg/mL	2.6–5.9	Human Serum	90.0–102	[65]
BNP	ZnCo_2_O_4_/N-CNTs-Ab/GCE	Amperometry DPV CV	0.01 pg/mL−1 ng/mL	3.34 fg/mL	2.9–3.5	Human Serum	97.0–102.1	[66]
cTnI	N, Zn-GQDs/GCE	DPV	10–10^6^ pg/mL	4.59 pg/L	9.09–11.1	Human Serum	92–97.1	[67]
cTnI	COOH-ZnONPs/GCE	EIS DPV	0.50 pM–3.3 × 10^5^ pM	1.04 pM	3.06–4.5	Human Serum	93.40−114.28	[68]
cTnI	CSA/MCH/Fc-COFNs-MBA/Au	CV DPV	10 fg/mL–10 ng/mL	2.6 fg/Ml	4.2	Human Serum	97.2–102.9	[69]
cTnI	PCN-AuNPs/LSGE	CV SWV	0.0001–1000 ng/mL	0.01 pg/mL	2.25	Human Serum	NR	[70]
cTnI	*p*CTAB/DES/Au-SPE and *p*CTAB/DES/Ab_2_/Au-SPE	DPV CV	0.04 ng/mL−50 ng/mL	0.0009 ng/Ml	0.37–1.94	Human Serum	NR	[71]
cTnI	N-prGO/COOH/PEG-aptamer/GCE	DPV	0.001–100 pg/L	1 pg/mL	4.3	Human Seum	98.2–101.7	[72]
cTnI	Fc-COOH-CIL-HCNTs/GCE	DPV	0.01–60 ng/mL	0.006 ng/mL	4.3–6.0	Human serum	96.4–103.3	[73]
cTnI	Ti disc/AuNPs/Apt	DPV	1–1100 pM	0.18 pM	3.28	Human serum	100.2–101.8	[74]
cTnI	DNA 3WJ/MB/Apt	CV	0 pM−100 nM	1.0 pM	NR	Human serum	NR	[75]
cTnI	MIP/BNQDs/GCE	DPV	0.01–5.0 ng/mL	0.0005 ng/mL	0.17–0.47	Human Plasma	NR	[76]
cTnT	N-MIP/SPCE	DPV	0.02–0.09 ng/mL	0.008 ng/mL	NR	Human Serum	NR	[77]
cTnT	cTnT-PANI/PMB/f-MWCNTs/ SPCE	DPV CV	0.10–8.0 pg/mL	0.040 pg/mL	1.3	Human Blood Plasma	91–112	[78]
NSE	AuNPs@MoS_2_/rGO/GCE	SWV EIS	0.01–1.0 pg/mL	3.00 fg/Ml	0.48	Human Serum	99.80–100.52	[79]
NSE	Ab_2_-Au/Fc@CuxO SPs/ HCNs-GR/GCE	Amperometry	500 fg/mL−100 ng/mL	25.7 fg/mL	4.6–7.6	Human Serum	87.8–95.4	[80]
NSE	Au–MoS_2_/MOF/GCE	SWV CV EIS	1.00 pg/mL−100.0 ng/mL	0.37 pg/mL	0.57–3.95	Human Serum	99–105.2	[81]
NSE	Ab/AuNPs/MES	CV DPV	1.0–750 ng/mL	0.34 ng/mL	3.1	Human Serum Saliva	NR	[82]
GDF15	MoS_2_/AuPtPd-Ab_2_/GCE	CV EIS	1.5 pg/mL−1.5 μg/mL	0.9 pg/mL	4.5	BSA	94.0–110.0	[83]
Ox-LDL	Mg–Fe_3_O_4_/PB/Ab/BSA MGCE	Chronoamperometry	10^−2^ μg/mL −10 μg/mL	9.80 × 10^−4^ μg/mL	2.10–4.95	BSA	NR	[84]
Gal-3	Ab_2_/AuNPs/MB/MSN/ GCE	DPV ASV	50 fg/mL−500 ng/mL	2.0 fg/mL	−2.8–4.6	Human Serum	95.2–107	[85]

NR: Not reported, RSD: Relative standard deviation, DPV: Differential Pulse Voltammetry, TNFα: Tumor Necrosis Factor Alpha, EIS: Electrochemical Impedance Spectroscopy CV: Cyclic Voltammetry, AuNPs: Gold Nanoparticles, MWCNTs: Multi-walled Carbon Nanotubes, IL-CS: Ionic Liquid (1-buthyl-3-methylimidazolium bis (trifluoromethyl sulfonyl)imide), ACV: Alternating Current Voltammetry, GCE: Glassy carbon electrode, IL6: Interleukin-6, TGFβ1: Transforming growth factor β1, AuNPs: Gold nanoparticles, SPCE: Screen-Printed Carbon Electrodes, NFs: Nanofibers, PAMAM: Polyamidoamine, AuE: Gold Electrode, ASPE: Poly-anthranilic acid, SPE: Graphite screen-printed electrode, ASPE: Poly-anthranilic acid, AuHCF: Layers of gold hexacyanoferrate, GPTES: 3-glycidoxypropyltriethoxysilane ITO: İndium tin oxide, PET: polyethylene terephthalate, Gal-3: Galectin-3, MIPs: Molecularly-imprinted polymers, TiO2NPs: Titanium-dioxide nano particles, N-GNRs-Fe-MOFs@AuNPs: N-doped graphene nanoribbons immobilized fe-based-Metal-organic frameworks deposited with Au nanoparticles, NT-proBNP: N-terminal B-type natriuretic peptid precursor, RTNs: Rough-surfaced trimetallic, cTnI: cardiac troponin I, Zn-GQDs: Zn co-doped graphene quantum dots, NT-proBNP: N-terminal pro-brain natriuretic peptide, ST2: Suppression of Tumorigenicity 2, GP: graphite paper, ChOx: Cholesterol oxidase, CSPPy-g-C3N4H+: Cylindrical spongy shaped polypyrrole, CRP: C-Reactive protein, ZnO/MPC/IL/anti-CRP/CPE: Porous carbon matrix/ionic liquid/C-reactive protein antibody/carbon paste electrode, PMPC-SH/SAM: Thiol-terminated poly (2-methacryloyloxyethyl phosphorylcholine)/self-assembled monolayer PEI-Fc: Fe (III) phthalocyanine, MB: Methylene blue, NH2-SWCNT: Aminoated single-walled carbon nanotubes, PCT: Procalcitonin SLG: Single-layer graphene, AgNp: Silver nanoparticle, BNP: Brain natriuretic peptide, NT-proBNP: N-terminal B-type natriuretic peptide precursor SWV: Square-wave voltammetry, LASV: Linear sweep anodic stripping voltammetry, SWASV: Square-wave anodic stripping voltammetry, N–ZnO NP: N-doped ZnO nanopolyhedra, PPIX: Protoporphyrin, IX N-CNTs: N-doped carbon nanotubes, Ab: Antibody-BNP, COOH-ZnONPs: Carboxylated ZnO nanoparticles (COOH-ZnONPs), Fc-COFNs: Ferrocene-based covalent organic framework nanosheets, CSA: cTnI specific aptamer, MCH: 6-mercapto-1-hexanol, PCN: Graphitic carbon nitride, LSGE: Laser-induced graphene electrodes, pCTAB: Cetyltrimethylammonium bromide, DES: Deep eutectic solvent, Ab2: Anti-CTnI polyclonal antibody, MoS2/rGO: Modified molybdenum disulfide and reduced graphene oxide, NSE: Neuron-specific enolase, Au/Fc@CuxO SPs: Rocene-functionalized cuprous oxide superparticles, HCNs-GR: Graphene supported by hollow carbon balls, MOF: The metal-organic framework MES: Microelectrode system, GDF15: Growth differentiation factor, cTnT: Cardiac troponin T, N-MIP: nano-molecularly imprinted polymer, N-prGO: Nitrogen-doped reduced graphene oxide, PEG: Polyethylene glycol, Apt: Aptamer, BNQDs: Boron nitride quantum dots, PANI: Polyaniline, PMB: Polymethylene blue, Ox-LDL: Oxidized low-density lipoprotein, MGCE: Magnetic glassy carbon electrode, BSA: Bovine Serum Albumin, ASV: Anodic Stripping Voltammetry, MSN: Mesoporous silica nanoparticles, LSV: Linear Sweep Voltammetry, Fc-ECG: A ferrocene derivative, MEL: Melamine, GE: Graphite electrodes, ERGO: Electrodes modified with electrochemically reduced graphene oxide, PTyr: polytyraminesolution, MPA: 3-mercaptoproponic acid, and PDMS: Polydimethylsiloxane.

## Data Availability

Not Applicable.
